# Short-Term Outcome of Unilateral Inspiration-Coupled Hypoglossal Nerve Stimulation in Patients with Obstructive Sleep Apnea

**DOI:** 10.3390/ijerph192416443

**Published:** 2022-12-08

**Authors:** Johannes Pordzik, Christopher Seifen, Katharina Ludwig, Tilman Huppertz, Katharina Bahr, Christoph Matthias, Haralampos Gouveris

**Affiliations:** Department of Otorhinolaryngology, Head and Neck Surgery, University Medical Center Mainz, 55131 Mainz, Germany

**Keywords:** obstructive sleep apnea, hypoglossal nerve stimulation, neurostimulation, outcomes

## Abstract

Hypoglossal nerve stimulation (HGNS) is a therapeutic option for patients with obstructive sleep apnea (OSA) and intolerance of positive airway pressure (PAP) therapy. Most reported data are based on multicentre pivotal trials with selected baseline core clinical features. Our aim was to investigate polysomnography (PSG)-based outcomes of HGNS-therapy in a patient cohort with higher average AHI and BMI than previously reported. Data of 29 consecutive patients (nine female; mean age: 55.52 ± 8.6 years, mean BMI 30.13 ± 3.93 kg/m^2^) were retrospectively evaluated. Numerical values of PSG- based metrics were compared before and after intervention using Wilcoxon’s rank-sum test. AHI (38.57/h ± 12.71, 24.43/h ± 13.3, *p* < 0.001), hypopnea index (24.05/h ± 9.4, 15.27/h ± 8.23, *p* < 0.001), apnea index (14.5/h ± 12.05, 9.17/h ± 10.86, *p* < 0.01), snoring index (262.68/h ± 170.35, 143.48/h ± 162.79, *p* < 0.001), cortical arousal index (20.8/h ± 10.34 vs. 14.9/h ± 8.36, *p* < 0.01) and cumulative duration of apnea and hypopnea during sleep (79.79 min ± 40.32 vs. 48.62 min ± 30.56, *p* < 0.001) were significantly lower after HGNS. HGNS provides an effective therapy option for selected patients not tolerating PAP-therapy with higher average AHI and BMI than usually reported. HGNS-therapy appears to suppress central nervous system arousal circuits while not eliciting peripheral autonomous sympathetic activation. Such metrics as the snoring index and the cumulative duration of respiratory events during sleep may be considered in future HGNS studies.

## 1. Introduction

Obstructive sleep apnoea (OSA) is the most common sleep-related disorder. It is characterized by repetitive partial or complete airway obstruction that occurs during sleep. OSA is associated with increased risk of hypertension [1], coronary artery disease [2], stroke [3], non-alcoholic hepatic steatosis [4], glaucoma [5], unfavourable oncologic outcome after therapy for head and neck squamous cell carcinoma [6] and further adverse effects. Progressive worsening of OSA results in poorer survival even after accounting for confounding factors [7]. Various options for OSA treatment are available including positional therapy (i.e., encouraging side sleeping), weight loss, mandibular advancement devices, positive airway pressure therapy (PAP), and surgery [8]. The standard first-line treatment for obstructive sleep apnea is continuous positive airway pressure (CPAP) delivered via various nasal or oronasal interfaces [9]. PAP therapy is very effective but not tolerated by some patients [10]. Hypoglossal nerve stimulation (HGNS) is an alternative treatment for patients with OSA failing or rejecting PAP therapy [11]. Selective electrostimulation of selected branches of the hypoglossal nerve and the first cervical motor nerve, increases activity of the genioglossal muscle, of the transverse and vertical tongue muscles and (usually) also of the geniohyoid muscle, resulting in protrusion of the stiffened tongue body [12]. Various devices are currently approved and available. One of them is the Inspire Medical System (Maple Grove, MN, USA), which is approved by the U.S. Food and Drug Administration (FDA) [13]. The Stimulation Therapy for Apnoea Reduction (STAR) Trial [11] showed a significant reduction in the apnea hypopnea index (AHI) and oxygen desaturation index (ODI) and led to FDA approval of the Inspire device. To date, most reported data are based on multicentre pivotal trials with strictly selected baseline core clinical features, such as AHI and BMI (body-mass index) [11,14]. Further data, especially from everyday clinical practice, are essential to evaluate the clinical outcome of hypoglossal nerve stimulation in a broader clinical context. The aim of this study was to evaluate additional parameters of polysomnography (PSG) after activation of the HGNS system. We hypothesized that standard PSG-based metrics would be significantly different after HGNS including the sleep architecture (as evidenced by sleep stage distribution) and the frequency of apnea and hypopnea, snoring, oxygen desaturation, periodic limb movements, arousals and sleep position. Additionally, we also investigated the potential impact of this neurostimulation therapy on peripheral autonomic activation.

## 2. Materials and Methods

### 2.1. Study Protocol

All consecutive patients (29) treated by unilateral inspiration-coupled hypoglossal nerve stimulation with implantation of the Inspire Medical System (Maple Grove, MN, USA) between February 2020 and June 2022 in our department of Otorhinolaryngology that is part of a tertiary university medical center were retrospectively included. All patients fulfilled the standard inclusion criteria for HGNS according to current guidelines [15], namely intolerance to PAP therapy, an apnea/hypopnea index (AHI) 15–65/h with <25% central apneas on cardiorespiratory polysomnography (PSG), BMI (body mass index) < 35 kg/m^2^, absence of complete concentric collapse on drug-induced sleep endoscopy (DISE) and absence of chronic neurodegenerative disease. The following relevant comorbidities at HGNS implantation time were observed: cardiovascular, such as arterial hypertension (in 14 patients) and coronary artery disease (in two), respiratory, such as asthma (in two patients) and chronic obstructive pulmonary disease less severe than GOLD 3 (in one), psychiatric, such as depression (in 7 patients), metabolic such as diabetes (in one patient). Average BMI of all patients was calculated. Full PSG type 1 was performed before and after implantation of the HGNS system according to the American Academy of Sleep Medicine (AASM) standard guidelines. A prelaryngeal microphone was used to detect snoring. All PSGs were evaluated visually and manually by sleep medicine experts. Postoperative PSG was performed 96.28 ± 27.02 days after activation of the implanted devices. The PSG report of each individual was analyzed for following parameters: AHI, total number of apneic events per hour (apnea index), total number of hypoxic events per hour (hypopnea index), longest duration of apnea and hypopnea events, cumulative time of apnea and hypopnea events, total number of snoring events per hour (snoring index), total number of oxygen desaturation events (≥4%) per hour (oxygen desaturation index), average oxygen desaturation in percentage, percentage of oxygen desaturation lower than 90% (t90), pulse variance index, maximal heart rate, mean heart rate, minimal heart rate, leg movement index (LM) (n/h), periodic limb movements index (PLM) (n/h), total sleep time in minutes (TST), percentage of N1, N2, N3 sleep, percentage of REM sleep, total number of arousal events per hour (arousal index), percentage of sleep time in upright, left, right, supine and abdominal position, AHI in supine position/not supine position.

The goal of this study was to compare retrospectively PSG-based sleep parameters before and after HGNS.

*Ethical statement*. All participants had provided informed consent to the use of their data for research purposes. The data were evaluated in a pseudonymized fashion. Due to this fact and the retrospective nature of the study, the local IRB (institutional review board) was consulted. A separate approval was waived by the local IRB, because all retrospective study procedures in this study were in accordance to local data protection and research practices. All procedures were in accordance with the Declaration of Helsinki.

### 2.2. Statistical Analysis

All data were statistically analyzed using SPSS 27 (IBM, Armonk, NY, USA). Categorical variables were described as number and percentage (%), and continuous variables were described as mean ± standard deviation. Comparisons between groups were analyzed using the Wilcoxon’s rank-sum test. Correlations were calculated according to Spearmann.

## 3. Results

Data from a total of 29 patients (nine female) were included. It turned out that mean age at the date of implantation was 55.52 ± 8.6 years and mean BMI was 30.13 ± 3.93 kg/m^2^.

**Figure 1:**
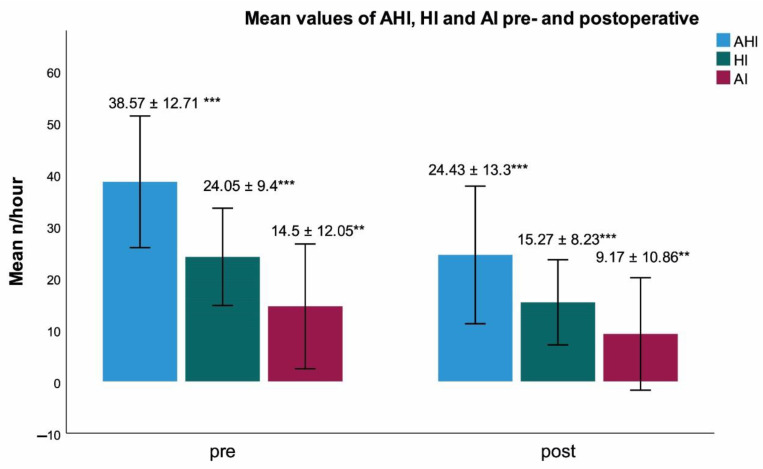
Comparison of AHI, HI and AI pre- and postoperative: AHI: 38.57 ± 12.71, 24.43 ± 13.3 *p* < 0.001, HI: 24.05 ± 9.4, 15.27 ± 8.23 *p* < 0.001, AI: 14.5 ± 12.05, 9.17 ± 10.86, *p* < 0.01, ** = statistically significant difference with *p* < 0.01; error bar with ± 1SD, *** = statistically significant difference with *p* < 0.001; error bar with ± 1SD.

**Figure 2:**
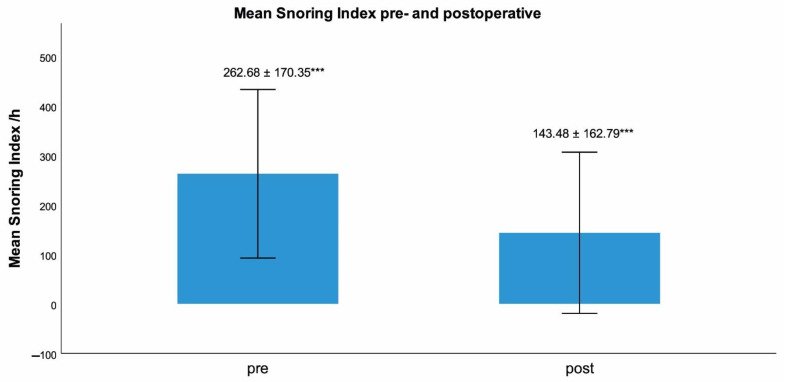
Comparison of snoring index pre-and postoperative: 262.68 ± 170.35, 143.48 ± 162.79, *p* < 0.001 *** = statistically significant difference with *p* < 0.001; error bar with ± 1SD.

The following respiratory parameters were significantly lower after HGNS (s. Table 1): AHI, apnea index, hypopnea index (s. Figure 1), cumulative duration of apnea and hypopnea, snoring index (s. Figure 2) and oxygen desaturation index (n/h) (*p* < 0.001, *p* < 0.01, *p* < 0.001, *p* < 0.001 and *p* < 0.001, *p* < 0.01, respectively). In addition, on average AHI was reduced by 37%, apnea index was reduced by 37%, hypopnea index was reduced by 37% (s. Figure 1), cumulative duration of apnea and hypopnea was reduced by 39%. Oxygen desaturation index (n/h) was reduced by 21%. No significant differences were found for the following PSG-based respiratory and cardiac/autonomic parameters: longest apnea (s), longest hypopnea (s), maximum heart rate (n/min), mean heart rate (n/min), minimum heart rate (n/min), pulse variance index, average oxygen saturation (%) and percentage of TST with oxygen saturation under 90% (t90, in %) (s. Table 1).

**Figure 3:**
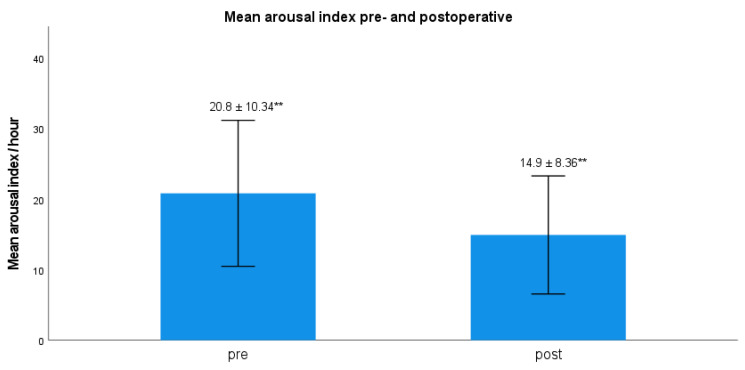
Comparison of arousal index pre-and postoperatively: 20.8 ± 10.34, 14.9 ± 8.36, *p* < 0.01 ** = statistically significant difference with *p* < 0.01; error bar with ± 1SD.

No significant differences were found regarding LM- and PLM-index (s. Table 1). The arousal index was significantly lower after HGNS (*p* < 0.01) (s. Figure 3). The percentage of cumulative apnea and hypopnea time TST adjusted was postoperatively significant reduced (s. Table 1). TST and the distribution of sleep stages, more precisely percentages of N1, N2, N3 and REM sleep distribution, the distribution of sleep position, more precisely percentages of upright, left, supine, right and abdominal position and the AHI in supine/other position did not differ significantly in both groups (s. Table 2).

No significant correlations were shown for AHI and cortical arousal index neither preoperative (correlation coefficient: 0.11, *p* = 0.85) neither postoperative (correlation coefficient: 0.21, *p* = 0.27).

## 4. Discussion

In this study, we demonstrated that respiratory distress, as witnessed by core PSG-based metrics, was significantly reduced after inspiration—coupled selective HGNS, even in the short term. Furthermore, we found that the cortical arousal index was significantly reduced after HGNS. No significant changes were observed neither for distribution of sleep stages nor for sleep position or of positional AHI.

It is well known that AHI is reduced after upper airway stimulation therapy. The STAR Trial [11] showed a significant reduction in the AHI by 52% and oxygen desaturation index (ODI) by 52% after 12 month including 124 patients. A pooled analysis of 584 patients, 126 in the STAR trial, 60 in the German cohort, 97 in the US cohort, and 301 in the ADHERE Registry (international registry: Data on adherence and outcome of upper airway stimulation) demonstrated an AHI reduction of 67% from 33.8 (15.5) events/h to 11.0 (13.6) events/h [16]. Some studies even described higher reduction in AHI up to 82% postoperative [14]. Our study also confirmed a significant reduction in AHI but showed a much lower reduction in AHI with 37% for AHI and 21% for ODI. However, previous studies had provided evidence that higher preoperative AHI was associated with higher therapy failure rate [17]. In accordance with such reports, it should be mentioned that preoperative AHI as well as preoperative BMI were higher in our study than in previous studies [11,14] (Table 3). This finding is original and provides further evidence for the effectiveness of HGNS therapy in patient cohorts with even higher average AHI and BMI.

In addition, we performed a further analysis (which was usually not performed in most existing reports) and found that the cumulative duration of apnea and hypopnea was also significantly reduced by over 30 min on average in our patient cohort. Furthermore, the percentage of cumulative apnea and hypopnea time TST was postoperatively significant reduced. We suggest that this specific feature should be further investigated in future studies because it provides evidence that HGNS therapy may have an even greater impact on the cumulative duration than on the frequency of respiratory events (apneas and hypopneas) during sleep. This argument is further supported by the fact that TST was not significantly different before and after HGNS therapy in our patient cohort (s. Table 1).

Notably, the snoring index was also significantly reduced in our patient cohort after HGNS therapy. Fewer data are available concerning snoring after HGNS therapy; one study proved a reduction in intrusive snoring based on partner report from 54% at baseline to 2% at 60 months [18]. Reporting outcomes on snoring after HGNS may be important because primary (habitual) snoring without concomitant OSA is associated with cardiovascular adverse effects including high blood pressure [19] and increased prevalence of carotid plaque [20], diabetes [21] and lipid abnormalities [22]. Furthermore, snoring leads to increased sleep fragmentation and poorer sleep quality of bedpartners [23]. The snoring index, but not the AHI, has also been positively correlated with the degree of non-alcoholic fatty liver disease (NAFLD) in patients with OSA [4]. Since the present study showed a quite significant reduction in the snoring index, we suggest that this PSG-based metric should be further investigated in future studies reporting results after HGNS therapy. Moreover, given the significance of habitual snoring in the social setting as well as the aforementioned comorbidities or cardio-metabolic risks associated with it, we suggest that future studies should be considered to evaluate use of HGNS in non-responders of other standard therapies for habitual snoring.

No significant effects of HGNS therapy on sleep architecture in patients with OSA were observed. Effects of upper-airway stimulation on sleep architecture have been investigated before, showing a reduction in N1-sleep after HGNS therapy [24,25]. In our cohort N1-sleep duration also tended to be shorter after HGNS-therapy.

Further, we showed a significant reduction in the cortical arousal index, in agreement with previous studies [24]. However, it should be mentioned that brainstem arousal and other minor changes in frequency might not be evident. Minimum, maximum and mean heart rate as well as pulse variance index did not show any significant differences. Pulse variance index is equal to pulse rate variability (PRV). PRV is obtained by pulse wave signals, such as the photoplethysmograms to refer to heart rate variability, which is measured via electrocardiographic signal [26]. Previously it could be shown, that heart variability index can estimate central efferent outflows [27] These observations have significant implications regarding sleep physiology in these patients, since HGNS therapy involving the tongue muscles during sleep could potentially cause an increase in the frequency of cortical arousals. However, this kind of unilateral inspiration-coupled neurostimulation therapy appears to suppress central nervous system arousal circuits while at the same time not eliciting any significant peripheral autonomous sympathetic activation. Suppression (or reduction) of the frequency of cortical arousals in these patients may be caused or associated to the reduction in the AHI as well as to the reduction in the cumulative duration of the respiratory events, namely apneas and hypopneas (s. Table 1).

On the contrary, the fact that no changes in peripheral sympathetic autonomic activation were observed in our patients, as attested by the absence of significant differences in minimum, maximum and mean heart rate as well as pulse variance index, may be due to the parallel absence of any significant differences on peripheral tissue oxygen supply, as evidenced by missing differences in average oxygen saturation and t90 in our cohort. In addition, these findings may also support the argument that this kind of neurostimulation provides stimulation of the tongue and upper airway muscle under the threshold for peripheral autonomic activation.

Arousals may be a more potent factor for sympathetic over-activity in OSA patients [28]. A significant association between arousals and increased odds of hypertension in patients with OSA has been reported [29]. Expectedly, any potential cardiovascular effects of this therapeutic modality may be mostly based on its effect on cardiorespiratory coupling and or on its effect on central nervous system arousal-circuits-associated cardiorespiratory control. Sleep disruption by OSA causes a postganglionic sympathetic nerve activation correlating with the breathing cycle. The peak intensity coincides with the arousal. Each apnea and its nadir are terminated with the subsequent hyperpnea [30,31,32].

In addition, no significant differences could be observed neither for the distribution of sleep positions through sleep nor for the ratio of AHI in supine position/AHI in non-supine position. One study so far investigated body position in upper airway stimulation for obstructive sleep apnea with comparable results to the ones in the present study [33]. Therefore, HGNS appears not to be as effective in positional as in non-positional OSA. Given that specifically patients on PAP-therapy with positional obstructive sleep apnea (POSA) or exclusive POSA (e-POSA) seem to have lower PAP therapy adherence [34], further investigations targeted to examine the effectiveness of HGNS specifically in patients with POSA and e-POSA appear necessary.

Our study included a relatively small sample size, and the data were acquired retrospectively. However, the quality and completeness of the gathered data were quite high. The postoperative data presented here involved the acute phase follow-up after 96.28 ± 27.02 days. It should be noted that there are few data in the literature for this acute phase after HGNS implantation. Most currently available data on HGNS therapy have been based on various multi-centered studies that led to approval of this therapeutic modality in different countries. Therefore, data based on everyday clinical practice are often missing. Our study aimed to provide outcomes of HGNS therapy in a standard everyday clinical practice. Even if the short-term postoperative outcomes presented in this report seem to be lower than presented before, we showed here that HGNS still leads to a very good clinical outcome in a patient cohort with higher AHI and BMI values than in the patient cohorts reported previously. Moreover, since a highly significant reduction in the snoring index was observed in our study, HGNS should be evaluated as a therapeutic option for patients with primary snoring not responding to other established forms of therapy. In addition, we suggest that the snoring index should be reported in future studies in OSA patients treated with this modality.

## 5. Conclusions

We demonstrated that standard core respiratory-related polysomnographic sleep parameters such as AHI, AI, HI and ODI were quite significantly reduced even in the short term after inspiration-coupled hypoglossal nerve stimulation therapy. Due to a higher average AHI and BMI values in our patient cohort, a tendency to lower reduction in these sleep parameters in our clinical practice was found, compared to other reported cohorts with lower average AHI and BMI. The cortical arousal index was significantly reduced, and hence hypoglossal nerve stimulation does not seem to cause arousals during sleep. No significant changes in positional AHI were found. A quite significant reduction in the snoring index as well as of the cumulative duration of respiratory events during sleep was shown.

## Figures and Tables

**Table 1:** Pre- and postoperative respiratory-associated PSG-based parameters.

	Preoperative	Postoperative	Comparison (*p*-Value)
AHI (n/h)	38.57 ± 12.71	24.43 ± 13.3	<0.001
Apnea index (n/h)	14.5 ± 12.05	9.17 ± 10.86	<0.01
Hypopnea index (n/h)	24.05 ± 9.4	15.27 ± 8.23	<0.001
Cumulative apnea and hypopnea time (min)	79.79 ± 40.32	48.62 ± 30.56	<0.001
Cumulative apnea and hypopnea time/TST-adjusted	0.21 ± 0.1	0.14 ± 0.08	<0.001
Longest apnea event (s)	43.79 ± 31.57	45.83 ± 26.42	0.57
Longest hypopnea event (s)	71.55 ± 33.08	62.9 ± 24.72	0.27
Snoring index (n/h)	262.68 ± 170.35	143.48 ± 162.79	<0.001
Maximum heart rate (n/min)	91.59 ± 10.46	92.14 ± 19.5	0.2
Mean heart rate (n/min)	63.03 ± 9.16	61.86 ± 8.95	0.27
Minimum heart rate (n/min)	48.97 ± 7.16	46.79 ± 9.56	0.14
Oxygen desaturation index (n/h)	36.89 ± 15.17	29.31 ± 15.65	<0.01
Average oxygen saturation (%)	92.93 ± 1.62	92.97 ± 1.74	0.82
t90 (%)	8.6 ± 10.24	6.28 ± 11.05	0.44
Pulse variance index (n/h)	27.37 ± 16.67	25.86 ± 17.56	0.44
LM (n/h)	45.63 ± 53.18	47.42 ± 57.14	0.65
PLM (n)	32.45 ± 43.89	30. 66 ± 44.86	0.77

**Table 2:** Sleep stages and sleep positions pre- and postoperative.

	Preoperative	Postoperative	Comparison (*p*-Value)
N 1 (%)	9.18 ± 6.83	8.64 ± 6.47	0.61
N 2 (%)	60.33 ± 11.45	59.57 ± 11.15	0.51
N 3 (%)	17.04 ± 7.93	18.59 ± 9.54	0.51
REM sleep (%)	12.44 ± 6.77	12.69 ± 8.09	0.8
TST (min)	372.23 ± 49.43	354.13 ± 52.39	0.22
Cortical arousal index (n/h)	20.8 ± 10.34	14.9 ± 8.36	<0.01
Upright position (%)	0.1 ± 0.56	0.79 ± 4.27	0.66
Right position (%)	33.9 ± 21.98	29.45 ± 17.83	0.29
Supine position (%)	35.62 ± 22.91	37.34 ± 26.62	0.79
Left position (%)	24.07 ± 18.08	26.14 ± 25.38	0.98
Abdominal position (%)	6.34 ± 14.23	6.14 ± 12.07	0.63
AHI in supine position /AHI in other position	3.05 ± 1.37	3.84 ± 3.4	0.61

**Table 3:** Demographic variables comparing main Hypoglossal Nerve Stimulation studies [14,16].

	Present Study	German	STAR	ADHERE	TJHU	UPMC
Age (years)	55.52 ± 8.6	57.6 ± 9.5	54.5 ± 10.2	59.2 ± 11.2	60.88 ± 11.12	62.84 ± 10.81
BMI (kg/m^2^)	30.13 ± 3.93	28.9 ± 3.5	28.4 ± 2.6	29.2 ± 3.8	29.29 ± 3.72	27.74 ± 3.66
Preoperative AHI (n/h)	38.57 ± 12.71	31.2 ± 13.2	32.0 ± 11.8	35.6 ± 15.3	35.88 ± 20.82	35.29 ± 15.33

## Data Availability

Please contact the authors for data requests.
